# Crowding Alone Cannot Account for Cosolute Effect on Amyloid Aggregation

**DOI:** 10.1371/journal.pone.0015608

**Published:** 2011-01-10

**Authors:** Shahar Sukenik, Regina Politi, Lior Ziserman, Dganit Danino, Assaf Friedler, Daniel Harries

**Affiliations:** 1 Institute of Chemistry, The Fritz Haber Research Center, The Hebrew University of Jerusalem, Edmund J. Safra Campus, Jerusalem, Israel; 2 The Fritz Haber Research Center, The Hebrew University of Jerusalem, Edmund J. Safra Campus, Jerusalem, Israel; 3 Department of Biotechnology and Food Engineering, The Russell Berrie Nanotechnology Institute, Technion - Israel Institute of Technology, Haifa, Israel; 4 The Russell Berrie Nanotechnology Institute, Technion - Israel Institute of Technology, Haifa, Israel; German Cancer Research Center, Germany

## Abstract

Amyloid fiber formation is a specific form of protein aggregation, often resulting from the misfolding of native proteins. Aimed at modeling the crowded environment of the cell, recent experiments showed a reduction in fibrillation halftimes for amyloid-forming peptides in the presence of cosolutes that are preferentially excluded from proteins and peptides. The effect of excluded cosolutes has previously been attributed to the large volume excluded by such inert cellular solutes, sometimes termed “macromolecular crowding”. Here, we studied a model peptide that can fold to a stable monomeric β-hairpin conformation, but under certain solution conditions aggregates in the form of amyloid fibrils. Using Circular Dichroism spectroscopy (CD), we found that, in the presence of polyols and polyethylene glycols acting as excluded cosolutes, the monomeric β-hairpin conformation was stabilized with respect to the unfolded state. Stabilization free energy was linear with cosolute concentration, and grew with molecular volume, as would also be predicted by crowding models. After initiating the aggregation process with a pH jump, fibrillation in the presence and absence of cosolutes was followed by ThT fluorescence, transmission electron microscopy, and CD spectroscopy. Polyols (glycerol and sorbitol) increased the lag time for fibril formation and elevated the amount of aggregated peptide at equilibrium, in a cosolute size and concentration dependent manner. However, fibrillation rates remained almost unaffected by a wide range of molecular weights of soluble polyethylene glycols. Our results highlight the importance of other forces beyond the excluded volume interactions responsible for crowding that may contribute to the cosolute effects acting on amyloid formation.

## Introduction

Amyloid aggregation is a specific form of protein self-oligomerization, which has been implicated in the pathogenesis of several neurodegenerative and other diseases [1]. The link to disease has made amyloids an intensive focus of research over the past decade [1], [2]. Currently, numerous proteins are known to undergo amyloid aggregation *in vivo* [3]–[5], and countless other proteins have been shown to form fibers *in vitro* under a variety of non-biological conditions [6]. It has, therefore, been hypothesized that the formation of amyloids is a general property common to many polypeptide chains [7].

Regardless of the identity of the aggregating protein, certain physical elements are shared by all known amyloid fibrils. Among these are a high β-sheet propensity and the ability to bind certain fluorescent dyes such as thioflavin T (ThT) [8]. While many details of fibril formation kinetics are yet unresolved, it is generally agreed that the aggregation process is initiated from a misfolded state [9], and proceeds via a nucleation-elongation mechanism [10]. Considerable effort has been aimed at controlling this often pathogenic process by, for example, adding ligands that are able to specifically bind fibrils in non-aggregating states [11], adding denaturing and stabilizing cosolutes [12], [13], applying hydrostatic pressure [14], or changing pH [15] as well as other solution conditions.

An important way to control amyloid aggregation is by the addition of cosolutes to solution. Cells contain a plethora of solutes occupying as much as 40% of their internal volume [16], a reality that is often ignored by *in-vitro* experiments [17]. Most of these cellular solutes do not interact specifically with proteins. They can, however, affect macromolecular thermodynamic stability through preferential exclusion from the protein surface, which raises the protein free energy in proportion to the preferentially excluding exposed surface area [18]. By folding and assuming more compact conformations with smaller interfacial area, proteins can lower this free energy penalty. This mechanism, often referred to as a “chemical chaperone” effect, may be sufficient to drive proteins to fold, bind, or shift towards aggregation [19]–[22]. The only requirement is the exclusion of cosolutes from the protein-water interface. Therefore, many chemically diverse molecules can act as chemical chaperones.

In order to quantify the chaperone effect on folding, it is common to follow the Wyman linkage [23] (or the Gibbs adsorption isotherm [24], [25]) that relates the extent of cosolute exclusion to the change in folding free energy ΔG with cosolute osmolality, *m_osm,_*:
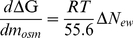
(1)


Here, *R* represents the gas constant, *T* the absolute temperature, and 55.6 the number of moles of water in 1 kg. Cosolute osmolality, *m_osm_*, is proportional to water's chemical potential when peptide concentrations are small. Finally, Δ*N_ew_* is the difference in the preferential hydration coefficient, representing the difference in the number of cosolute excluding water molecules between the folded and unfolded states. For cosolutes that are preferentially excluded from the unfolded D state more than the compact N state, Δ*N_ew_* is negative. Therefore, by virtue of their net repulsion from protein surfaces, cosolutes can shift the equilibrium state of proteins towards more compact states [26], [27].

Various models have related the size of cosolutes to Δ*N_ew_*. For example, modeling the excluded volume of crowders using scaled particle theory (SPT) has shown that the shifts in macromolecular equilibrium in the presence of these cosolutes correlates well with experimental values [28]. These models assume that as cosolutes grow in volume their steric exclusion becomes stronger. Thus, there is a larger entropic gain to peptide folding in the presence of larger cosolutes due to the greater reduction in volume excluded from cosolutes upon folding. As a result, 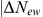 increases, and more cosolute excluding water molecules are “released” to the bulk upon protein folding.

Here, we focus on the effect of excluded cosolutes on protein aggregation processes. One large, chemically diverse class of protein-stabilizing cosolutes, composed of many different molecules, is sometimes termed “macromolecular crowders”. These may include proteins, nucleic acids, carbohydrates or other large molecules that do not interact directly or specifically with protein molecules (or other macromolecules of interest). Studies of the effect on amyloid aggregation of large molecular crowders, such as the hydrophilic polysaccharide ficoll or polyethylene glycols (PEGs), have shown an increase in the propensity of proteins to form aggregates, and that the lifetimes for both fibril nucleation and elongation were shorter in the presence of crowders, with a correlation to crowder size and concentration [20], [28], [29].

Another class of protein stabilizing cellular cosolutes is often termed “compatible osmolytes”. These are small molecules used by the cell to counteract environmental stress, and include free amino acids and their derivatives, urea derivatives, and polyhydric alcohols [16]. The effect of osmolytes is often measured in terms of the osmotic pressure that these molecules exert. It has been shown that macromolecular crowding can, in many cases, be equivalently described in terms of osmotic pressure [30], [31], since in these cases both effects are mediated by steric or excluded volume interactions, and are therefore closely related. Interestingly, however, several recent experiments on amyloid forming systems have shown that, unlike macromolecular crowders, compatible osmolytes such as trehalose [32] and inositol [33] are able to inhibit amyloid aggregation. This apparent discrepancy awaits resolution.

**Figure 1:**
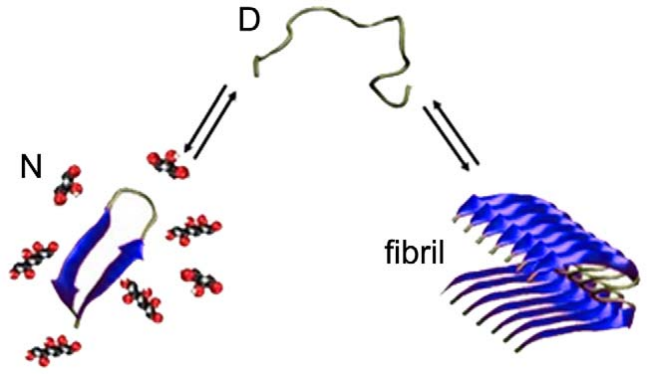
Schematic of MET16 states and transitions. In buffered environment (pH 7) the peptide exists in two-state equilibrium between native (N) and unfolded (D) conformations. After ∼90 min a third, fibrillar aggregate conformation appears. The folded conformations appearing in the fibril need not be the same as N.

In this study, we followed the effect of macromolecular crowders and compatible osmolytes on the folding and aggregation of a model peptide (termed here MET16, see methods for details) that was developed and extensively studied by Searle and coworkers [34], [35]. We focused on polyethylene glycols (PEGs) of various molecular weights (representing large, inert macromolecular crowders) as well as the smaller polyol osmolytes sorbitol and glycerol. MET16 displays at least 3 distinct resolvable states in solution, shown schematically in Fig. 1. At short times (less than 90 minutes) in buffered solution (pH 7) MET16 is found in either an unfolded (D, Fig. 1) or native (N, Fig. 1) state, with a folding free energy under those conditions of 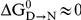. It has previously been shown that this dynamic equilibrium can be shifted towards the β-hairpin state by adding chemical chaperones [34], [36]. We found that the intramolecular folding reaction of MET16 was stabilized in a size and concentration dependant manner for all cosolutes used here.

At longer times, a third, aggregated state of MET16 appears in solution (fibril, Fig. 1), and the peptide undergoes amyloid-like fibril formation, reaching a steady-state within roughly 24 hours. Using ThT fluorescence and CD spectroscopy measurements, we followed the rates of MET16 folding and aggregation. Surprisingly, the aggregation process was affected differently by the presence of the two classes of cosolutes. The polyol osmolytes substantially slowed the nucleation process and increased the fraction of monomers that had undergone fibrillation in a concentration dependant manner. However, regardless of size or concentration, all PEGs we used showed no considerable effect on the aggregation kinetics of the peptide.

Our findings contrast other known modulations of aggregation kinetics by cosolutes that have been explained in terms of excluded volume mechanisms that are entropically driven. We suggest that the possible mechanisms of action of these so-called “inert” cosolutes in biological systems may involve previously neglected forces in solution. Specifically, ranking excluded solutes according to their size alone is insufficient to predict their effectiveness in promoting or slowing aggregation. A more detailed knowledge of solution structure as well as peptide-water-cosolute interactions is needed.

## Results

### Monomer hairpin conformation is stabilized by cosolutes

**Figure 2:**
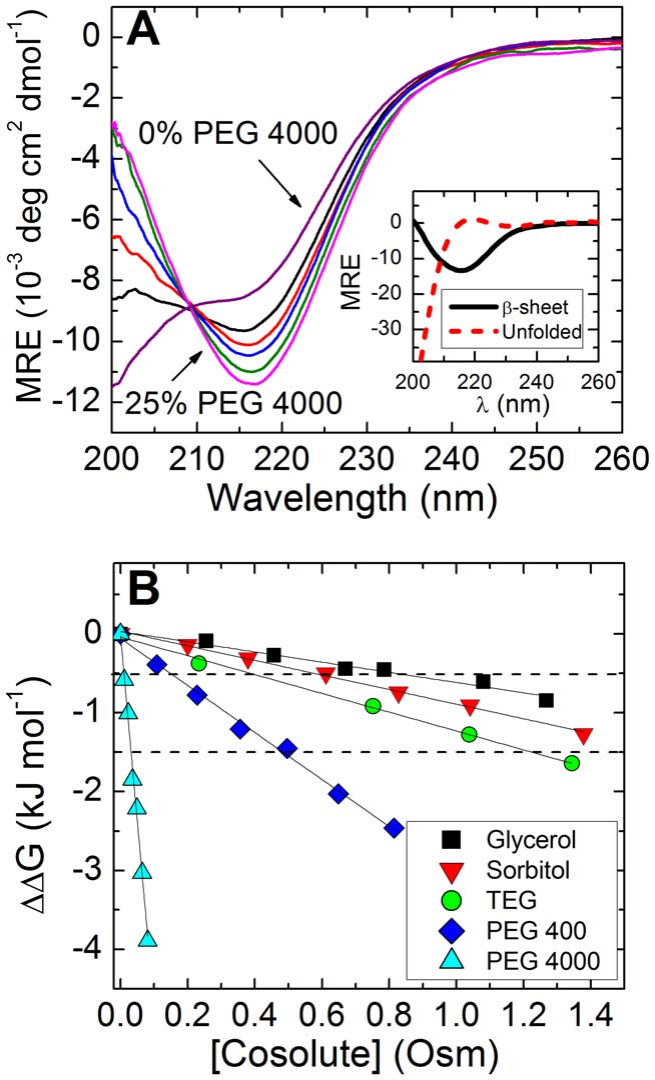
Effect of cosolutes on monomeric peptide structure and stability. (**A**) Representative CD spectra of MET16 with increasing PEG 4000 concentrations (0, 8, 12, 17, 21, and 25% (w/w)). Inset shows CD spectra for fully folded MET16 in the presence of 55% (w/w) methanol (solid line) and the calculated unfolded peptide spectra (dashed line, see text for details). (**B**) Peptide folding free energy (ΔΔG) as a function of cosolute concentration. The dashed lines delineate cosolute concentration corresponding to ΔΔG = −0.5 kJ/mol and −1.5 kJ/mol.

To measure the impact of cosolutes on the unassociated monomer equilibrium (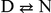, Fig. 1), CD spectra of MET16 were measured in the presence of different solutes. Fig. 2A shows spectra obtained in the presence of increasing PEG 4000 concentrations. The spectra showed an apparent isodichroic point at wavelength λ = 209 nm for all cosolute concentrations, suggesting that the peptide primarily exists in two distinct states. CD and NMR experiments have previously shown that the peptide exists in either the β-hairpin (N) or unfolded (D) states, with a free energy change for folding 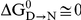
for the transition between the states at pH 7 and T = 298 K [34]–[36].

Our data allowed to evaluate the equilibrium constant for monomer folding/unfolding using CD, as previously extensively validated and demonstrated by Searle and coworkers, as well as in our work [34], [36], by comparing the mean residue ellipticity (MRE) minimum at λ = 215 nm to the MRE of the unfolded and fully folded peptide at the same wavelength. The limiting value for the ellipticity of the fully folded conformation was determined by the peptide signal at 55% (w/w) MeOH. The limiting value for the unfolded conformation was previously shown by NMR experiments and by examining the spectra of an unfolded random amino acid sequence to have an ellipticity of zero at this wavelength [35]. Furthermore, the fully folded spectrum obtained experimentally could be weighted and subtracted from the spectrum of MET16 at equal peptide and buffer concentrations to obtain the unfolded spectrum. The two basis spectra derived for the N and D states are shown in the inset of Fig. 2A. Interestingly, the unfolded state basis spectrum is very similar to that expected from the poly (L-proline)-type (P_II_) conformation [37], [38]. This P_II_ motif has been implicated as the prevailing form of other well known amyloid formers, such as Aβ1-28, in the monomeric unaggregated state [39], [40]. In fact, multiple evidence suggests that P_II_ is an important intermediary in amyloid formation. Accumulating evidence points to P_II_ as a possible “killer conformation” that leads to the formation of ordered aggregates such as fibrils, while more “random” coils give rise to amorphous aggregates [41].

Using this model, we could determine 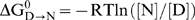, where [N] and [D] represent the concentrations of native and non-native structures. These concentrations are related to the fractions of native and unfolded states through 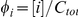 where φ represents the mole fraction, the index *i* represents N or D (native and unfolded states respectively), and 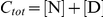 is the total molar concentration of the peptide, as measured by UV-Vis absorbance.

Fig. 2B shows the change in folding free energy upon cosolute addition, 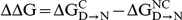, as a function of cosolute concentration. The linear dependence of ΔΔG on cosolute concentration seen in Fig. 2B implies that the difference in number of water molecules excluded to the cosolute between folded and unfolded peptide conformations is constant, as indicated by Eq. 1, and that this number increases with cosolute size.

### Aggregation kinetics of MET16 is cosolute-dependant

We next followed the effect of cosolutes at various concentrations on peptide aggregation using ThT fluorescence. To compare reactions where the initial folded monomer populations are equal, we equated ΔΔG for each reaction mixture using appropriate cosolute concentrations from both PEG and polyol groups. The β-hairpin content at different cosolute concentrations can be predicted by interpolation of data points in Fig. 2B, and verified by CD, thereby allowing us to compare solutions where MET16 monomer population are stabilized equally (same ΔΔG) with different cosolutes. This creates an equal peptide stabilizing effect in the presence of the different cosolutes, and gives an equal starting point in terms of peptide folded/unfolded monomer population for the aggregation reactions.

**Figure 3:**
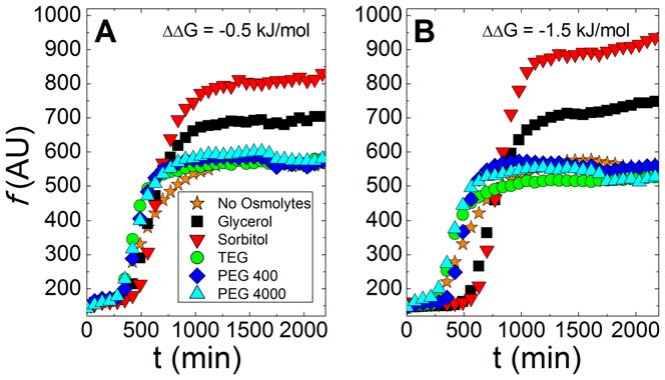
Kinetics of amyloid fibril formation followed by ThT fluorescence. ThT emission signal recorded at λ = 485 nm vs. time from initiation of the aggregation reaction, in the presence of different cosolutes. Emission is proportional to the amount of monomers that had undergone fibrillation. Cosolute concentration was chosen so that all reactions began with the same folded peptide content, with a change in folding free energy of (*a*) ΔΔG = −0.5 kJ/mol, and (*b*) ΔΔG = −1.5 kJ/mol. Cosolute concentrations (in w/w%) were for (*a*) glycerol 7.03, sorbitol 9.53, TEG 4.31, PEG 400 5.62, PEG 4000 3.97; (*b*) glycerol 17.90, sorbitol 23.21, TEG 11.12, PEG 400 16.22, PEG 4000 11.21. Some intermediate data points were omitted for clarity.

Fig. 3 shows ThT fluorescence emission, *f*, as a function of time for aggregation reactions, where the initial folded peptide conformation was stabilized by cosolutes to ΔΔG = −0.5 kJ/mol (φ_N_ = 0.6, Fig. 3A) and −1.5 kJ/mol (φ_N_ = 0.7, Fig. 3B), as represented by dashed lines in Fig. 2B. Reactions were initiated by adding phosphate buffer to the unbuffered solution and followed by measuring fluorescence emission at λ = 485 nm over time. The resulting curves were fit to a sigmoidal function using the lag time for the aggregation, *t_lag_*, the fibril elongation lifetime, *τ_el_*, and the ThT emission plateau (maximum) value 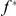
(see Methods for further details). The values for *t_lag_* are presented in Table 1. Fits show that the initial lag time became increasingly longer in a cosolute concentration dependent manner in the presence of glycerol, and even more so in sorbitol. In contrast, regardless of PEG length and concentration, the presence of PEGs did not significantly alter the nucleation kinetics compared to the reaction in the absence of cosolutes.

**Table 1:** Aggregation lag times *t_lag_* (in minutes) for different peptide stabilities ΔΔG (in kJ/mol).^a^

ΔΔG	−0.5	−1.0	−1.5	−2.5
Glycerol	390±50	470±50	540±30	750±30
Sorbitol	490±40	520±20	640±40	900±80
TEG	290±30	200±30	240±10	270±10
PEG 400	350±40	410±70	370±20	490±40
PEG 4000	300±30	280±20	270±20	300±30

a value of *t_lag_* in the absence of cosolutes was 340±80 min.

The elongation phase follows nucleation and is characterized by a sharp increase in ThT emission. The slope in the plot of *f* vs. time for this stage (Fig. 3A and B) represents the rate of monomer addition to the fibril, as characterized by the apparent elongation rate, 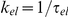. We found that, regardless of cosolute type or concentration, *τ_el_* does not deviate from the experimental error range set in the absence of cosolutes, as seen in Table 2. The ThT emission signal plateaus when the system reaches steady state, suggesting that the rate of monomer addition is equal to the rate of monomer dissociation. The emission signal at this point (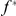) is related to the amount of monomers that have undergone fibrillation, with a higher signal representing a larger aggregated peptide population.

**Table 2:** Elongation lifetime *τ_el_* (in minutes) for different peptide stabilities ΔΔG (in kJ/mol).^a^

ΔΔG	−0.5	−1.0	−1.5	−2.5
Glycerol	100±8	100±10	110±4	120±20
Sorbitol	120±40	100±20	88±5	150±30
TEG	70±11	81±5	84±4	59±1
PEG 400	62±3	50±5	58±7	53±3
PEG 4000	90±20	59±9	70±30	53±3

a value of *τ_el_* in the absence of cosolutes was 100±40 min.

We noted, however, that fluorescence signals may be highly sensitive to solution environmental conditions, such as temperature and viscosity. These artifacts can make it complicated to compare the absolute intensity for different solutions. We have verified that ThT emission values may vary up to 30% in pure water versus at the highest cosolute concentrations (see Methods for details). Therefore, we only considered signal changes that are higher than this error margin to indicate a substantial variation in emission of fibril-bound ThT. In addition, there was no significant increase in ThT emission due to cosolutes in the absence of MET16, suggesting that ThT emission is related only to MET16 and not to cosolute presence (see Methods for details). We could, therefore, determine that in the presence of the polyols, glycerol and to a larger extent sorbitol, there was a significant increase in the amount of monomer that had undergone fibrillation at equilibrium, as seen in Table 3. In contrast, PEGs showed no significant change in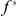, regardless of length and concentration, as compared to the reaction in the absence of cosolutes.

**Table 3:** Fluorescence emission plateau f^*^ (in AU) for different peptide stabilities ΔΔG (in kJ/mol).^a^

ΔΔG	−0.5	−1.0	−1.5	−2.5
Glycerol	690±43	755±17	725±11	731±18
Sorbitol	781±51	878±56	901±13	1073±14
TEG	566±57	561±12	515±8	451±18
PEG 400	572±48	569±27	558±18	482±13
PEG 4000	584±46	559±20	536±21	564±40

a value of *f* in the absence of cosolutes was 560±20.

### Similar fibrils are formed in the presence and absence of cosolutes

**Figure 4:**
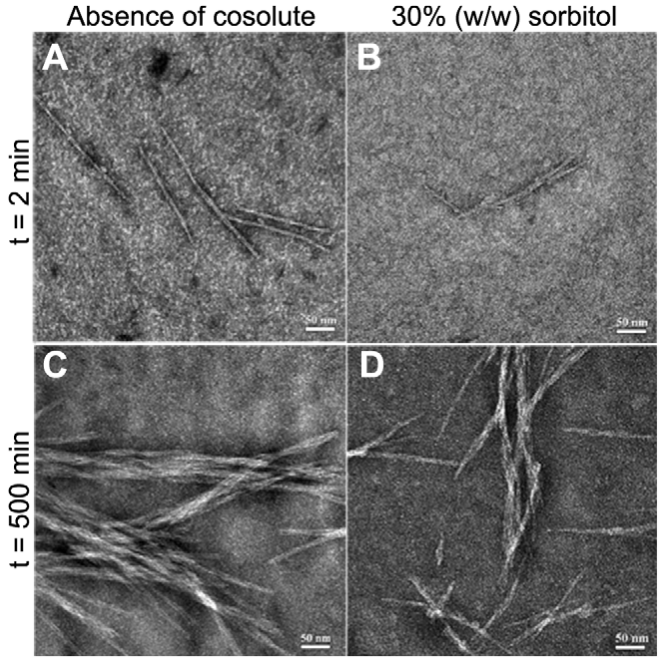
TEM images of MET16 fibrils at different times and under different solution conditions. Negative stain TEM images taken from aggregation mixtures at different times: After 2 minutes, in the absence (*a*) and presence (*b*) of 1.5 M sorbitol, and after 500 minutes, in the absence (*c*) and presence (*d*) of 1.5 M sorbitol.

To validate that MET16 forms amyloid fibrils, as suggested by the increase of ThT fluorescence signal, fibrillation reactions were imaged using negative-stain transmission electron microscopy (TEM). Fig. 4 shows micrographs taken at the beginning of the aggregation reaction, and after incubation for 500 minutes in the presence and absence of 30% (w/w) sorbitol. The micrographs show similar morphologies, both in the presence and absence of sorbitol. This supports the fluorescence measurements that showed signals typical of amyloid fibrils. In addition, the existence of similar morphologies indicates that the presence of cosolutes did not significantly change fibril structure.

**Figure 5:**
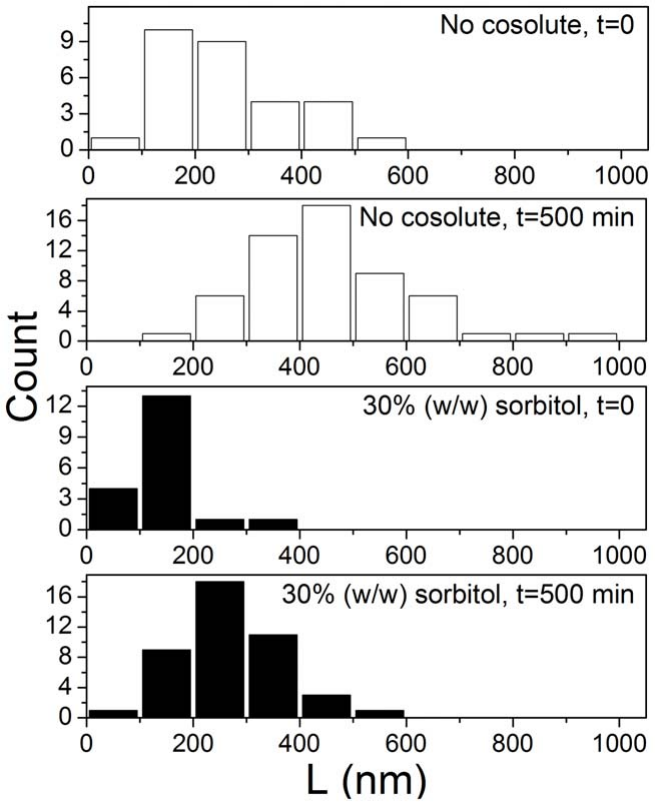
Length distribution analysis of fibrils imaged using TEM. Fibril lengths were measured in the absence of cosolutes, (**A**) at *t* = 0 (average length, as calculated directly from measurements, 263±114 nm), (**B**) at *t* = 500 min, (average length 458±146 nm); and in presence of 30% (w/w) sorbitol, (**C**) at *t* = 0 min (average length is 142±64 nm), (**D**) at *t* = 500 min (average length 265±95 nm). Errors in average length are standard deviation of length measurements.

Length distribution analysis was performed on the micrographs taken from *t* = 0 and *t* = 500 min, in the presence and absence of sorbitol. Each distribution represents an average taken from several micrographs of the same grid. Fig. 5 shows the length distribution of fibers, revealing similar starting length distribution for fibrils in both reactions. However, as the reaction progressed, the fibrils in the absence of sorbitol grew by a greater length than those in the presence of the osmolyte. The fibrils in aqueous solution grew on average by a length ΔL≈194 nm over the course of 500 minutes, whereas those in a solution containing 30% (w/w) sorbitol grew by a length ΔL≈124 nm over the same time.

### Cosolutes modify fibrillation reaction rates, but do not affect the final conformation of fibrils

**Figure 6:**
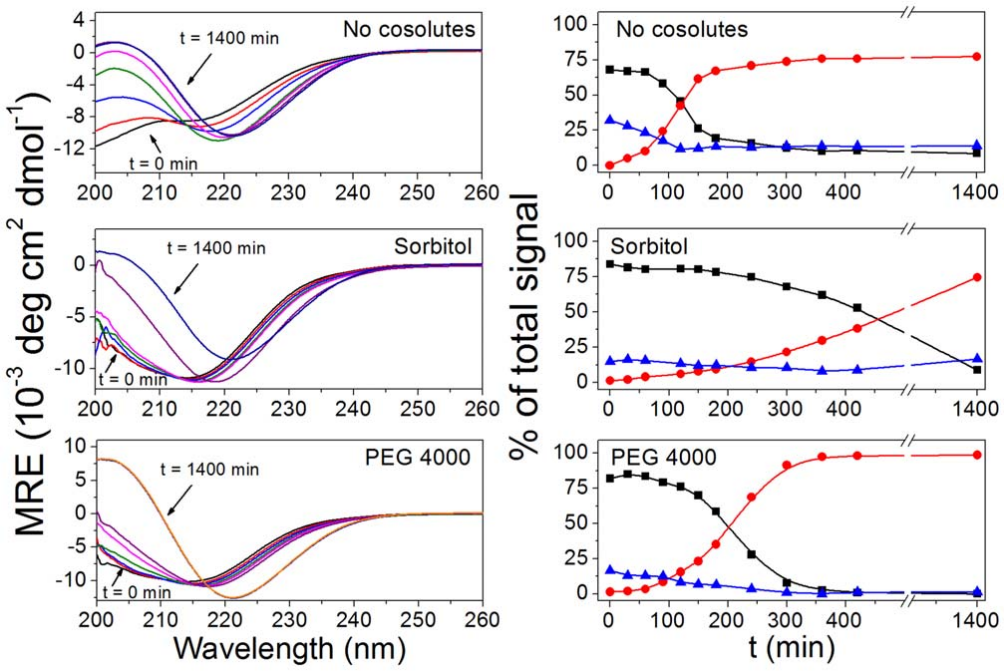
Kinetics of amyloid formation followed by CD spectroscopy. (***left column***) CD spectra measured at different times of the aggregation process in the absence (*top*) and presence of sorbitol (*center*) and PEG 4000 (*bottom*). (***right column***) Contribution of unfolded (*triangles*), β-sheet (*squares*) and amyloid (*circles*) formations to each of the CD spectra presented on the left column, as determined by CCA analysis, shown as a function of time for each of the aggregation reactions shown on the left. Lines are guides for the eyes.

To assess the changes in MET16 secondary structure over the course of aggregation we used CD spectroscopy in the presence of various cosolutes leading to equal populations of the monomer hairpin conformation φ_N_ at *t* = 0. Fig. 6, *left* shows spectra acquired over 1400 min for MET16 aggregation reactions in the absence of cosolutes and in the presence of sorbitol or PEG 4000 at a concentration corresponding to ΔΔG = −2.5 kJ/mol (or to φ_N_≈0.8 at *t* = 0). The minimum seen at λ = 215 nm in the presence of osmolytes at short times compared with the sample with no cosolute reflects the higher population of the β-hairpin form in the presence of both sorbitol and PEG 4000.

As the reaction progressed (*t*≈200 min) spectra showed a decrease (in absolute value) and a red-shift in the ellipticity at the minimum, from λ = 215 nm towards λ = 220 nm, concurrent with the abolishing of the isodichroic point. These observations indicate that at least one additional peptide state is formed in solution, and that simple two-state equilibrium can no longer be assumed. The formation of fibrils is a likely cause of these changes, as also suggested by ThT and TEM results that show aggregate formations at similar times.

To further quantify and assign CD features to the transition from monomer to amyloid, the spectra of each reaction were deconvoluted into base components by using the convex constraint analysis (CCA+) algorithm, developed and described in detail elsewhere [42]. Unlike other CD analysis methods, which require a dataset of pre-determined protein spectra set, CCA requires no additional input other than the experimental set itself. This makes CCA better suited for the analysis of short proteins and peptides that do not contain distinct or ubiquitous structured domains. The CCA+ algorithm was used on a data matrix containing all time-resolved spectra of a single CD aggregation experiment in order to find a user-defined number of base components (*P*) (see additional details in Materials and Methods). These base components can be linearly combined to re-form (simulate) the experimental spectra.

**Figure 7:**
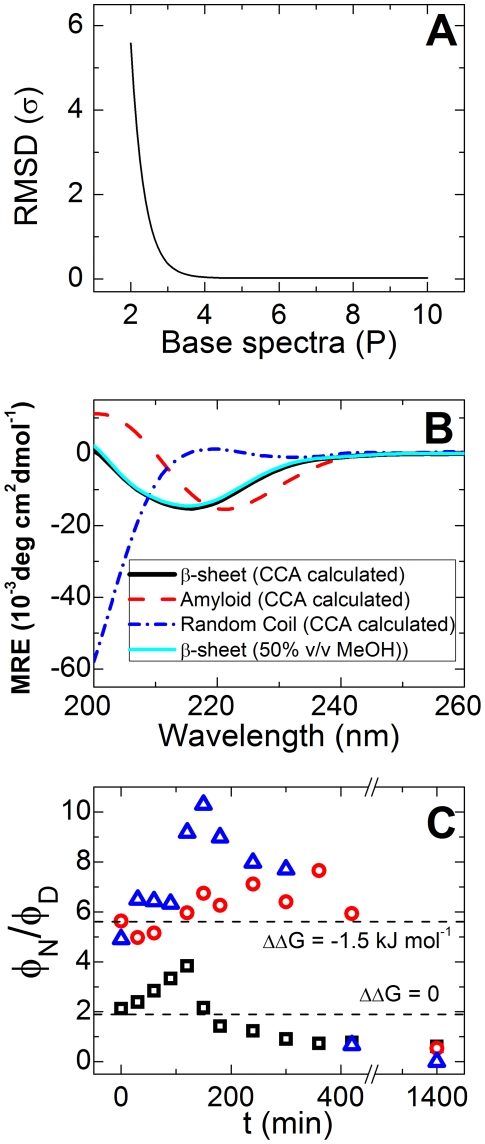
Analyses of CD-resolved kinetics. (**A**) RMS deviation of simulated CD spectra based on the derived basis set of P spectra (**B**) Simulated CD curves of the three basic structures calculated by CCA. These correspond to pure β-sheet (solid black line), amyloid (dashed) and unfolded (dot-dashed). The solid cyan line is the experimental spectrum of the fully folded peptide in the presence of 55% (w/w) MeOH. (**C**) Ratio of β-sheet (φ_f_) over unfolded (φ_u_) mole fraction as a function of time for MET16 in water (*squares*), and enough sorbitol (*circles*) or PEG 4000 (*triangles*) to induce a stabilization of ΔΔG = −1.5 kJ/mol to the β-sheet conformation. The dotted lines represent theoretical values for the equilibrium constant for folding 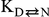
for the reaction in aqueous media (ΔΔG = 0) and in the presence of the cosolutes (ΔΔG = −1.5 kJ/mol).

To find the optimal number of components, the experimental spectra were deconvoluted into increasing numbers of base spectra, and the root-mean-square (RMS) deviation, σ, of the original set from those simulated using the determined base spectra was evaluated. The optimal number of base components for the data was found to be *P* = 3, after which there was only minor improvement in σ, as seen in Fig. 7A. One of the three determined base components corresponded closely to N, the β-hairpin signal as previously evaluated, while another component had a molar ellipticity close to 0 at λ = 215 nm, which fits the known form of unfolded peptides and is reminiscent of the P_II_ motif [43]. This base spectrum was thus assigned to the D state, representing the unfolded peptide conformation. These two spectra may be linearly combined to closely match the experimental spectrum at *t* = 0 in all three reactions presented in Fig. 6. We therefore suggest that the remaining third component corresponds to the amyloid morphology. The three base components are shown in Fig. 7B.

Fig. 6, *right* shows the contribution of each base component to the total experimental spectra as a function of time, as determined by the CCA+ analysis. As expected for short times, cosolutes shifted the population of the peptide towards the more compact conformation compared with their value in pure water (folded mole fraction φ_N_≈0.8 with sorbitol and PEG 4000 compared to φ_N_≈0.6 without osmolytes). As the reaction evolved, there was a decrease in both unfolded and β-hairpin conformations, while the contribution of the amyloid-associated component increased. The ratio of β-hairpin to random coil contribution, which should remain constant for a quick pre-equilibrium step, seems to drop dramatically in the experiments after about 400 minutes, especially in the presence of cosolutes (see Fig. 7C). We suggest that this may be due to the limits of sensitivity of the measurement for low monomer concentration (and hence low signal for the N and D components).

The reaction reached equilibrium at *t* 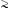
1000 min, with the amyloid component as the major contributor to the spectrum. It is apparent that though the final, amyloid-rich spectrum of each reaction was similar, with a minimum at λ = 221 nm, the rate for the accumulation of this component varied for the different solutions. In the absence of osmolytes, amyloids formed fastest, while in the presence of PEG the process was only slightly slowed. However, sorbitol showed a significant prolonging of amyloid formation. This result corresponds to the longer *t_lag_* measured in the presence of sorbitol in the ThT fluorescence experiments, Fig. 2.

### Viscosity does not account for the observed changes in aggregation kinetics

**Figure 8:**
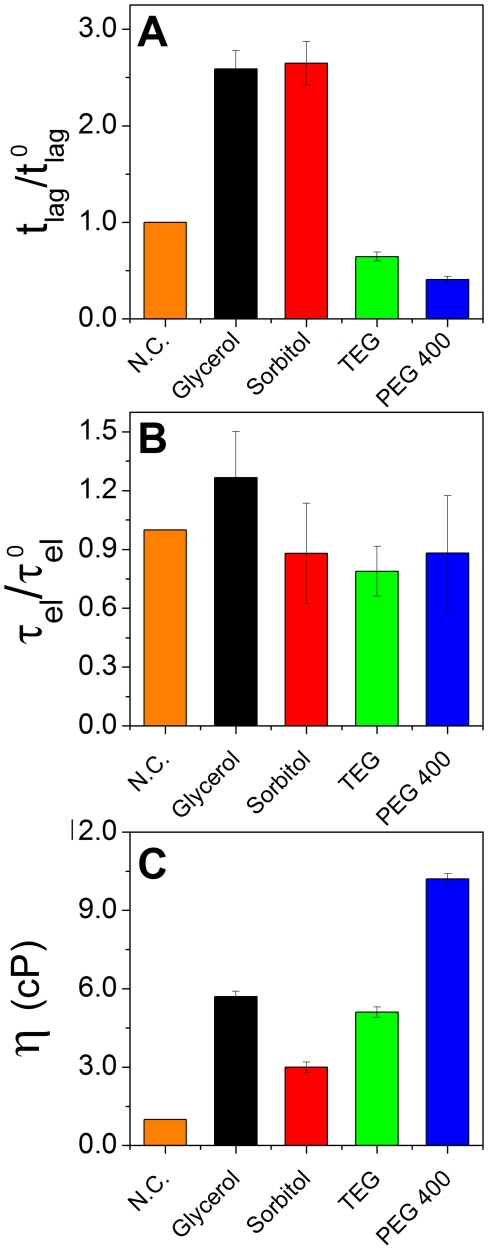
Effects of high viscosity on amyloid formation kinetic constants. Relative lag times *t_lag_* (**A**) and elongation lifetimes *τ*_el_ (**B**) for 50% (w/w) glycerol, TEG and PEG 400, and 30% (w/w) for sorbitol. N.C. corresponds to the reaction in the absence of cosolute, to which the other reactions are compared. (**C**) The viscosity of each solution, in cP.

The effect that cosolutes have on solution viscosity is a potential source for altered association kinetics in solution, and subsequent amyloid formation. Specifically, when reactions are diffusion limited, high viscosity may cause a slowing of the observed association kinetics. To assess the influence of bulk viscosity, *η*, on reaction kinetics, we measured ThT fluorescence for 3 reactions containing 50% (w/w) of glycerol, TEG and PEG 400, and a reaction in the presence of 30% (w/w) of sorbitol. Under these conditions, PEG 400 has the highest bulk measured viscosity, glycerol and TEG have similar viscosities, and sorbitol has the lowest, as shown in Fig. 8C. Sorbitol and glycerol, despite their low viscosities, had the largest effect on extending *t_lag_* (∼2.5 fold increase), while TEG and PEG 400 appear to shorten *t_lag_* (Fig. 8A). The small decrease in *t_lag_* for the ethylene glycols compared to aggregation in pure water may be an artifact attributed to changes in fluorescence emission in the presence of high cosolute concentrations, and is within experimental error. However, the increase in *t_lag_* for glycerol and sorbitol is significant. The large difference between the viscosity of the polyols and the ethylene glycols that inversely correlate with nucleation times indicates that the kinetic effects observed cannot be attributed solely to solution viscosity. Moreover, *τ_el_* appears to be almost independent of viscosity, as the nucleation time in the presence of the cosolutes falls within error range of that in the absence of cosolutes in all three cases (Fig. 8B).

## Discussion

Our aim in this study was to determine the effect of various excluded cosolutes on amyloid formation. We have used ThT fluorescence, TEM, and CD spectroscopy to follow the aggregation process of a β-hairpin folding peptide into amyloid fibers in the presence of different cosolutes at different concentrations. As we discuss below, we have found that the effect of cosolutes depends not only on their preferential exclusion from folded *versus* unfolded peptides, but also on their chemical identity.

### Cosolutes effects vary at different stages of the aggregation

At short times, the addition of cosolutes acts to stabilize the compact hairpin conformation of the monomeric peptide in a concentration dependant manner (Fig. 2B). This effect correlates well with the molecular size of the cosolute, so that the large PEGs exert a strong stabilizing force on the 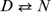
reaction (ΔΔG≈47 kJ/mol M^−1^ for PEG 4000), while polyols show a weaker stabilizing effect on the peptide at the same concentrations. This effect has previously been reported for both small osmolytes [44], [45] and larger, macromolecular crowders [46]. By obtaining slopes of ΔΔG vs. concentration (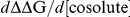, sometimes called *m*-values for proteins [47]) for all cosolutes used in this work, a similar magnitude of folded conformation stabilization could be induced by using different cosolutes at the appropriate concentrations.

To follow amyloid formation, a pH jump (at *t* = 0) was used to initiate aggregation. Differences in kinetics were revealed in the presence of the two cosolute types and in the absence of cosolutes. While nucleation times, *t_lag_*, slowed with cosolute concentration in the presence of the polyols (up to 2.5-fold for ∼30% (w/w) sorbitol), PEGs showed a negligible effect on the nucleation process, as shown in Fig. 3 and Table 1. This suggests that the difference in the chemical nature of cosolutes may play an important part in their action on the various peptide conformations present in solution. Namely, the monomeric conformations could be affected differently than aggregates by the various cosolutes. This point has been theoretically demonstrated by applying the Kirkwood-Buff theory of solutions to a system containing cosolutes and an aggregating protein [48].

Examination of the cosolute molecular structure shows that, among several differences in their interactions, polyols can form multiple hydrogen bonds with the surrounding water molecules as both acceptors and donors [49] while PEGs can only act as hydrogen bond acceptors, perhaps leading to the difference in their ability to act as “chemical chaperones” [50]. The difference in the effect of polyols on the monomeric folding and aggregation reactions can be rationalized if we consider how each state the peptide assumes is affected differently by the presence of osmolytes, for example due to a different capacity of each to form hydrogen bonds. In this way the osmolytes may destabilize the aggregating nucleus in favor of the monomeric state, while concurrently acting to stabilize the fibril once it is formed. This can explain the observation that sorbitol and glycerol increased nucleation time, but also caused more monomers to undergo fibrillation at equilibrium, as seen in Fig. 3.

It is interesting that the unfolded monomer D conformation shows a CD spectral signature close to the P_II_ helix conformation, implicated as a possible amyloid forming intermediate [41]. We suggest that this flexible structure that lacks intrachain hydrogen bonds, and is fully hydrated in aqueous solution, can be significantly impacted in aqueous osmolyte solutions that are known to alter the hydrogen bonding network [49], [51], [52].

Regardless of cosolute type or concentration, changes in elongation lifetime, *τ_el_*, did not vary significantly compared to those in the absence of cosolutes. This insensitivity may in part reflect the large error inherent to these measurements, especially for the reaction in aqueous solution (*τ_el_* = 100±40 min). However, experiments performed under high cosolute concentrations and high viscosity (Fig. 8) reveal that even under those extreme conditions the elongation process is almost unaltered by cosolutes. In addition, following elongation, the ThT emission plateau value, 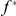, increased in the presence of polyols (up to a 2-fold increase for sorbitol), but remained unaltered relative to the aqueous solution in the presence of PEGs (Fig. 3 and Table 2).

The height of the emission plateau, 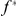 can be related to the extent of fibril formation, and hence to the equilibrium constant of fibril formation. The equilibrium constant, in turn, is linked to the ratio of monomer addition and monomer detachment rates. Therefore, taken together, the insensitivity of *τ_el_* to the addition of cosolutes with concurrent variation in 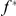 implies that it is the rate of peptide monomer detachment from the fibril that is modified in the presence of cosolutes. This would be possible if the rate-limiting step in the MET16 aggregation process, by which the peptide attaches to the growing fibril, is not diffusion limited.

Supporting this finding, recent experiments and simulations show that in their addition to the fibril, peptides must undergo slow structural modifications in order to assimilate into the fibril. This mechanism is consistent with a so-called dock-lock mechanism for amyloid fibril formation [53], [54]. It is, therefore, possible that solution viscosity does not affect the elongation rates because it does not alter the rate-limiting step in the dock-lock mechanism. The detachment process, on the other hand, may still be affected by osmolytes, as the changes in 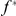 suggest. Indeed, it has been shown that for other associating macromolecules, excluded cosolutes can strongly modulate binding association kinetics primarily by affecting the off-rate rather than the on-rate [55], [56].

### Cosolute size correlates with MET16 intramolecular folding but not with intermolecular association

All cosolutes we have tested showed a linear dependence of folding ΔΔG with cosolute concentration. Furthermore, the extent of intramolecular folding of the MET16 monomer correlates well with cosolute size. Specifically, the slope of ΔΔG versus cosolute concentration is proportional to the change in the number of cosolute excluding water molecules in the folding process (Eq. 1) [31]. Indeed, the large PEG 4000 has an *m*-value that is over an order of magnitude greater than that of glycerol.

Models based on scaled particle theory (SPT) that have been used to predict the extent of molecular crowding, typically consider cosolutes as hard bodies, and use their dimensions to estimate the thermodynamic and kinetic effect caused by the resulting confinement in their presence [57], [58]. This model was shown to be applicable to the kinetics of the aggregation process of proteins such as apolipoprotein II, which was more rapid in the presence of crowders in a concentration dependent manner [28]. More recently the same model was applied to the fibrillation of a wide range of amyloid forming proteins, showing a cosolute size-dependant increase in fibril elongation rates [59]. A different model used Brownian dynamics to show that although the reaction rate may be enhanced by the presence of crowding agents, decreased diffusion constants can cause a slowing of association reactions [60]. Unlike SPT, this model predicts the reaction rates will not increase monotonically with crowder size. Instead, there is a more complex dependency on both the size and the total volume occupied by the crowder.

However, we found that MET16 aggregation kinetics in the presence of cosolutes cannot be simply accounted for using steric exclusion considerations alone. While the small polyols, glycerol and sorbitol, slowed the rate of nucleation, the larger PEG 400 and 4000 showed little or no effect on the aggregation process. Interestingly, while sorbitol's effect was substantial, TEG that has approximately the same molecular size as sorbitol (partial molar volumes of 129.29±0.08 cm^3^/mol for TEG [61] and 119.16 cm^3^/mol for sorbitol [62]), and whose effect on monomer folding was comparable to that of sorbitol (*m*-value = −1.8 kJ/mol compared to −1.3 kJ/mol for sorbitol, Fig. 2B), showed little or no effect on the aggregation process.

Our findings point to a fundamental difference in the effects of the polyols (glycerol and sorbitol) and PEGs. Because all the cosolutes used in this work are found to be preferentially excluded from the peptide in these concentration ranges (as indicated by the negative Δ*N_ew_* see Eq. 1, and Fig. 2B.), we suggest that the difference in the action of these cosolutes could result from the different effect that polyols have on aggregated forms versus any pre-aggregated forms. The details of the attractive and repulsive interactions acting between all the components in this ternary system, water, peptide, and cosolutes, and their modification for different folding states leading to fibril formation are not resolved. This makes it difficult to predict how the association reaction will be affected. Moreover, the structure of the intermediates is not resolved, making it hard to estimate how the final equilibrium will be shifted according to Eq. 1. However, because cosolutes show net exclusion from the peptide, it is reasonable to assume that interactions responsible for the altered aggregation kinetics are mediated by properties of the bulk aqueous solution surrounding the peptide [49], [51].

Crowding models discuss the decrease in preferential hydration as a result of folding driven by increase in cosolute translational entropy. Osmolytes, while possibly having a similar entropic contribution to folded peptide stabilization, have in addition been shown to modify the folding free energy by creating net repulsive interactions with the peptide backbone compared to those formed with water [44], [63], [64]. Our previous results on the monomer MET16 folding show that, in contrast to the crowding mechanism that is purely entropic, osmolytes can also act through adding a favorable enthalpic driving force for further folding [36]. While the molecular origin of this enthalpic contribution is still unclear, experimental evidence suggests that water structuring forces at the protein interface could be involved. These interfacial waters can be regarded as “poorer solvents” for osmolytes, causing a depletion of the osmolytes from the protein surface [65]. This exclusion due to poor solubility stems from the interaction of the osmolytes with water molecules, perhaps explaining the enthalpic nature of the interactions [63], [66]. Simulations and experiments [49], [51] indicate that one way in which polyols and sugars may be able to exert an enthalpic effect to folding is by inducing a change in the water hydrogen bond network upon solvation. This change may alter peptide-solution interactions, which would in turn affect the peptide conformations according to their exposure to the solution. These interactions require extensive additional theoretical analysis.

To conclude, we have demonstrated that peptide amyloid aggregation can be affected differently by two chemically different families of cosolutes. We found that for the MET16 peptide, polyols slowed aggregation kinetics, while larger PEGs showed no effect. This impact may depend on peptide identity, and underscores the importance of solvent–mediated cosolute-peptide interactions for the different aggregation precursors, as well as for the amyloid structure. Specifically, our experiments suggest that molecular crowding cannot account for the full effect of cosolutes on protein aggregation kinetics. It would be interesting to link the action of osmolytes on peptide folding and aggregation to their effect on interactions in solution, as well as to peptide sequence and properties.

## Materials and Methods

### Materials

Sorbitol, triethyleneglycol (TEG), and polyethyleneglycol (PEG) 400 and 4000 were from Fluka AG (Bucks, Switzerland). Glycerol was from Frutarom (Haifa, Israel). All chemicals were used without further purification. MET16 peptide (sequence Ac-KKYTVSINGKKITVSI-OH) was from GL Biochem (Shanghai) Ltd. (Shanghai, China). Peptide identity was confirmed using an ABI Voyager MALDI TOF mass spectrometer and peptide purity was checked using a Merck-Hitachi analytical HPLC system and was shown to be over 98% pure. Peptide stock concentration was measured using a Shimadzu UV1650-PC UV/Vis spectrophotometer by absorbance of a single tyrosine residue (λ = 274 nm, ε = 1490 M^−1^cm^−1^). Peptide stock was kept in purified water at −70°C until ready for use. Experiments were conducted in phosphate buffer, pH 7, at concentrations between 10 and 50 mM as specified below, in the absence of any additional salt.

### Solution properties measurements

Osmolality was obtained using a Vapro 5520 vapor pressure osmometer (Wescor, Inc., Logan UT). Samples were taken immediately after ThT fluorescence measurements and pipetted from the plate to the osmometer chamber. Measurements were performed in triplicates. The instrument was calibrated using 100, 290 and 1000 mOsm standards every 20 measurements. The room was kept at an ambient temperature of 25°C.

Viscosity was measured using a Gilmont falling ball type viscometer (Gilmont Instruments, Barington, IL) with a steel or glass ball. The density of the osmolyte solutions was obtained from previously published data [67], [68]. Prior to measurement, the instrument was washed several times with water, followed by a wash with the solution to be tested. The solution was filtered through a 4.5 µm frit into the tube, and degassed under vacuum for 10 minutes. The solution was allowed to equilibrate with a 25°C thermostated water bath for an additional 10 minutes. The time it takes the ball to complete the fall was measured using a calibrated stop-watch, and repeated in quadruplets.

### Transmission electron microscopy

Samples for imaging were prepared by removing aliquots from an ongoing aggregation reaction of 100 µM MET16 in 10 mM phosphate buffer, with and without the presence of 30% (w/w) sorbitol at different times during the reaction. All images were analyzed using ImageJ software (NIH, Bethesda MD) [69]. Fibril length analysis was performed on a set of at least 7 micrographs from each reaction time, containing a total of 148 unbundled fibrils whose length could be measured. Bundles that were too thick to quantify or fibrils with varied thickness were omitted since their lengths could represent several misaligned fibrils.

Negative stained samples were prepared as described previously [70], by placing a carbon coated grid on a 15 µl sample drop for 2 min, blotted with filter paper, chemically stained with 2% uranyl acetate for 2 min, blotted again, and air dried. Negatively stained specimens were examined in a transmission electron microscope, FEI T12 G^2^, operated at 120 kV. Images were recorded digitally on Gatan UltraScan 2k×2k CCD camera with the DigitalMicrograph software package.

### Circular dichroism

CD spectra of the peptide were recorded on a JASCO J-810 Spectrophotometer (JASCO, Japan) using the supplied SpectraManager software. The temperature was kept constant at 25°C using a temperature controlled water bath. Samples were made fresh from stock before each measurement. 400 µL samples containing 100 µM MET16 peptide and 10 mM phosphate buffer, together with various concentrations of different osmolytes were placed in 0.2 cm quartz cells (Starna, CA) and spectra were recorded in the wavelength range λ = 195–260 nm, with 5–10 accumulations for each measurement and a data pitch of 0.1 nm. The cuevette was inverted before each measurement to minimize loss of signal due to precipitation of aggregates below the spectrometer beam. Background CD spectra of the appropriate buffer and cosolutes were recorded and subtracted from each spectrum.

### CCA Analysis

The CD spectra, obtained as described over the time-course of MET16 aggregation, were compiled to a data matrix with the use of the CCA+ software, publically available from http://www.chem.elte.hu/departments/protnmr/cca/. In addition to those spectra, the matrix contained an experimentally derived spectrum obtained in the presence of 50% (w/w) methanol, where the monomeric peptide has been shown to be fully in the N state, and the calculated unfolded spectrum of MET16. The unfolded state spectrum was obtained by calculating φ_N_ and φ_D_ prior to aggregation (see Fig. 2B and corresponding text) and subtracting the weighted, fully-folded spectrum from the MET16 spectrum in the absence of any cosolutes, at *t* = 0. The matrix was then deconvoluted using the CCA module as previously described [42], [71], yielding a predefined number, *P*, of base spectra components.

### ThT fluorescence assay

Assay solutions contained 10 µM MET16 peptide, 3 µM ThT and 50 mM Phosphate buffer, with various concentrations of osmolytes as indicated. Each sample was run in quadruplicates at the least. A volume of 100 µL was transferred into each well of a 96-well plastic plate (Nunc, NY) and sealed using a transparent adhesive seal. The plate was loaded to a TECAN GenIos PRO plate reader at 25°C. The plate was shaken at 100 rpm for 60 seconds, and allowed to rest for an additional 30 seconds before each measurement. Fluorescence intensity *f* was measured at 10–30 min intervals with excitation at λ = 450 nm and emission at λ = 485 nm, for a total time of 48–72 hours. The resulting aggregation curve was fit to a sigmoidal of the form:
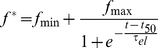
(2)


Where 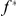 = *f_max_*−*f_min_* is the peak ThT emission, τ_el_ the apparent elongation lifetime, *t_50_* is the time required to reach half of the maximal emission, and the lag time for nucleation is *t_lag_* = *t_50_*-2*τ_el_*. Note that this is a phenomenological fit of the data and does not necessarily represent the actual kinetic mechanism of fibrillation.

**Figure 9:**
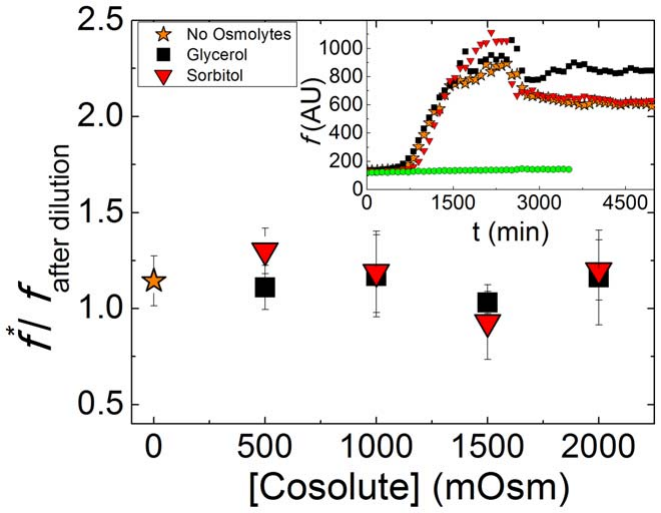
Effects of cosolute addition on ThT fluorescence. Ratio of ThT emission values at λ = 485 nm before and after cosolute addition. A value close to 1.0 represents no change in emission upon dilution. Inset shows ThT fluorescence emission vs. time, with the point of dilution at *t* = 2600 minutes. The value of signal at the plateau prior to dilution (f^*^) (Eq. 2) was divided by the average emission value of the hour following ThT addition to obtain the relative deviation values of the fluorescence at peak emission as a result of cosolute addition. Circles (*green*) show the emission of buffered ThT without the addition of MET16.

In order to assess the solvent effects on 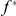, an aggregation reaction was first allowed to run to equilibrium in the presence of 3 µM ThT and 50 mM phosphate buffer. At this point, a solution containing 50 mM phosphate buffer, 3 µM ThT and a high concentration of each cosolute, detailed in the caption in Fig. 2B, is added to the reaction mixture. The effect of the cosolute was tested by calculating the peak ThT emission 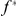 at steady state prior to dilution (evaluated using the sigmoidal fit shown in Eq. 2) and then immediately following cosolute addition. A deviation of 30% at most was seen for the different osmolytes, as shown in Fig. 9.

## References

[1] Chiti F, Dobson CM (2006). Protein misfolding, functional amyloid, and human disease.. Annu Rev Biochem.

[2] Haass C, Selkoe DJ (2007). Soluble protein oligomers in neurodegeneration: lessons from the Alzheimer's amyloid β-peptide.. Nat Rev Mol Cell Biol.

[3] Harper JD, Lansbury PT (1997). Models of Amyloid Seeding in Alzheimer's Disease and Scrapie: Mechanistic Truths and Physiological Consequences of the Time-Dependent Solubility of Amyloid Proteins.. Annu Rev Biochem.

[4] Glenner GG (1980). Amyloid Deposits and Amyloidosis - the β-Fibrilloses.. N Engl J Med.

[5] Dobson CM (1999). Protein misfolding, evolution and disease.. Trends Biochem Sci.

[6] Izhack C, Ehud G (2008). Amyloids: Not Only Pathological Agents but Also Ordered Nanomaterials.. Angew Chem.

[7] Dobson CM (2003). Protein folding and misfolding.. Nature.

[8] LeVinne HI (1993). Thioflavine T interaction with synthetic Alzheimer's disease β-amyloid peptides: Detection of amyloid aggregation in solution.. Protein Sci.

[9] Calamai M, Chiti F, Dobson CM (2005). Amyloid Fibril Formation Can Proceed from Different Conformations of a Partially Unfolded Protein.. Biophys J.

[10] Morris AM, Watzky MA, Finke RG (2009). Protein aggregation kinetics, mechanism, and curve-fitting: A review of the literature.. Biochim Biophys Acta.

[11] Xu YC, Shen JJ, Luo XM, Zhu WL, Chen KX (2005). Conformational transition of amyloid β-peptide.. Proc Natl Acad Sci U S A.

[12] Ignatova Z, Gierasch LM (2006). Inhibition of protein aggregation in vitro and in vivo by a natural osmoprotectant.. Proc Natl Acad Sci U S A.

[13] Natalello A, Liu J, Ami D, Doglia SM, de Marco A (2009). The osmolyte betaine promotes protein misfolding and disruption of protein aggregates.. Proteins Struct Funct Bioinformat.

[14] De Felice FG, Vieira MNN, Meirelles MNL, Morozova-Roche LA, Dobson CM (2004). Formation of amyloid aggregates from human lysozyme and its disease-associated variants using hydrostatic pressure.. FASEB J.

[15] Picotti P, De Franceschi G, Frare E, Spolaore B, Zambonin M (2007). Amyloid fibril formation and disaggregation of fragment 1–29 of apomyoglobin: Insights into the effect of pH on protein fibrillogenesis.. J Mol Biol.

[16] Yancey PH, Clark ME, Hand SC, Bowlus RD, Somero GN (1982). Living with water stress: evolution of osmolyte systems.. Science.

[17] Ellis RJ (2001). Macromolecular crowding: an important but neglected aspect of the intracellular environment.. Curr Opin Struct Biol.

[18] Zimmerman SB, Minton AP (1993). Macromolecular Crowding - Biochemical, Biophysical and Physiological Consequences.. Annu Rev Biophys Biomol Struct.

[19] Zimmerman SB, Trach SO (1988). Effects of macromolecular crowding on the association of E.coli ribosomal particles.. Nucleic Acids Res.

[20] Munishkina LA, Ahmad A, Fink AL, Uversky VN (2008). Guiding Protein Aggregation with Macromolecular Crowding.. Biochemistry.

[21] Minton AP (2000). Implications of macromolecular crowding for protein assembly.. Curr Opin Struct Biol.

[22] Minton AP (2001). The influence of macromolecular crowding and macromolecular confinement on biochemical reactions in physiological media.. J Biol Chem.

[23] Wyman J, Anfinsen J CB, Anson ML, Edsall JT, Frederic MR (1964). Linked Functions and Reciprocal Effects in Hemoglobin: A Second Look..

[24] Adrian Parsegian V, Thomas Z, Wilfred DS (2002). Protein-water interactions.. International Review of Cytology: A survey of Cell Biology;Molecular Mechanisms of Water Transport across Biological Membranes:.

[25] Gibbs JW, B HA, vN RG (1993). On the equilibrium of heterogeneous substances;.

[26] Harries D, Rosgen J (2008). A practical guide on how osmolytes modulate macromolecular properties.. Biophysical Tools for Biologists: Vol 1 In Vitro Techniques.

[27] Rosgen J (2007). Molecular basis of osmolyte effects on protein and metabolites.. Methods Enzymol.

[28] Hatters DM, Minton AP, Howlett GJ (2002). Macromolecular crowding accelerates amyloid formation by human apolipoprotein C-II.. J Biol Chem.

[29] Munishkina LA, Cooper EM, Uversky VN, Fink AL (2004). The effect of macromolecular crowding on protein aggregation and amyloid fibril formation.. J Mol Recognit.

[30] Parsegian VA, Rand RP, Rau DC, Michael LJ, Gary KA (1995). Macromolecules and water: Probing with osmotic stress..

[31] Parsegian VA, Rand RP, Rau DC (2000). Osmotic stress, crowding, preferential hydration, and binding: A comparison of perspectives.. Proc Natl Acad Sci U S A.

[32] Liu F-F, Ji L, Dong X-Y, Sun Y (2009). Molecular Insight into the Inhibition Effect of Trehalose on the Nucleation and Elongation of Amyloid β-Peptide Oligomers.. J Phys Chem B.

[33] McLaurin J, Golomb R, Jurewicz A, Antel JP, Fraser PE (2000). Inositol stereoisomers stabilize an oligomeric aggregate of Alzheimer amyloid β peptide and inhibit abeta -induced toxicity.. J Biol Chem.

[34] Maynard AJ, Sharman GJ, Searle MS (1998). Origin of β-Hairpin Stability in Solution: Structural and Thermodynamic Analysis of the Folding of a Model Peptide Supports Hydrophobic Stabilization in Water.. J Am Chem Soc.

[35] Griffiths-Jones SR, Maynard AJ, Searle MS (1999). Dissecting the stability of a β-hairpin peptide that folds in water: NMR and molecular dynamics analysis of the β-turn and β-strand contributions to folding.. J Mol Biol.

[36] Politi R, Harries D (2010). An enthalpic mechanism for peptide stabilization by protecting osmolytes.. Chem Commun.

[37] Shi Z, Woody RW, Kallenbach NR, George DR (2002). Is polyproline II a major backbone conformation in unfolded proteins?.

[38] Sreerama N, Woody RW (1994). Poly(Pro)II Helixes in Globular Proteins: Identification and Circular Dichroic Analysis.. Biochemistry.

[39] Syme CD, Blanch EW, Holt C, Jakes R, Goedert M (2002). A Raman optical activity study of rheomorphism in caseins, synucleins and tau.. Eur J Biochem.

[40] Eker F, Griebenow K, Schweitzer-Stenner R (2004). Aβ1–28 Fragment of the Amyloid Peptide Predominantly Adopts a Polyproline II Conformation in an Acidic Solution.. Biochemistry.

[41] Blanch EW, Morozova-Roche LA, Cochran DAE, Doig AJ, Hecht L (2000). Is polyproline II helix the killer conformation? a raman optical activity study of the amyloidogenic prefibrillar intermediate of human lysozyme.. J Mol Biol.

[42] Perczel A, Hollosi M, Tusnady G, Fasman GDF (1991). Convex constraint analysis: a natural deconvolution of circular dichroism curves of proteins.. Protein Eng.

[43] Siligardi G, Drake AF (1995). The importance of extended conformations and, in particular, the PII conformation for the molecular recognition of peptides.. Biopolymers.

[44] Auton M, Bolen DW (2005). Predicting the energetics of osmolyte-induced protein folding/unfolding.. Proc Natl Acad Sci U S A.

[45] Stanley CB, Strey HH (2008). Osmotically Induced Helix-Coil Transition in Poly(Glutamic Acid).. Biophys J.

[46] Stagg L, Zhang S-Q, Cheung MS, Wittung-Stafshede P (2007). Molecular crowding enhances native structure and stability of α/βprotein flavodoxin.. Proc Natl Acad Sci U S A.

[47] Fersht AR (1999). Structure and Mechanism in Protein Science..

[48] Gee MB, Smith PE (2009). Kirkwood—Buff theory of molecular and protein association, aggregation, and cellular crowding.. J Chem Phys.

[49] Guo F, Friedman JM (2009). Osmolyte-Induced Perturbations of Hydrogen Bonding between Hydration Layer Waters: Correlation with Protein Conformational Changes.. J Phys Chem B.

[50] Welch WJ, Brown CR (1996). Influence of Molecular and Chemical Chaperones on Protein Folding.. Cell Stress Chaperones.

[51] Politi R, Sapir L, Harries D (2009). The Impact of Polyols on Water Structure in Solution: A Computational Study.. J Phys Chem A.

[52] Kuffel A, Zielkiewicz J (2010). The hydrogen bond network structure within the hydration shell around simple osmolytes: Urea, tetramethylurea, and trimethylamine-N-oxide, investigated using both a fixed charge and a polarizable water model.. The Journal of Chemical Physics.

[53] Cannon MJ, Williams AD, Wetzel R, Myszka DG (2004). Kinetic analysis of β-amyloid fibril elongation.. Anal Biochem.

[54] Nguyen PH, Li MS, Stock G, Straub JE, Thirumalai D (2007). Monomer adds to preformed structured oligomers of Aβ-peptides by a two-stage dock-lock mechanism.. Proc Natl Acad Sci U S A.

[55] Sidorova NY, Rau DC (2000). The dissociation rate of the EcoRI-DNA-specific complex is linked to water activity.. Biopolymers.

[56] Gurnev AP, Harries D, Parsegian VA, Sergey MB (2009). The Dynamic Side of the Hofmeister Effect: A Single-Molecule Nanopore Study of Specific Complex Formation.. ChemPhysChem.

[57] Minton AP (2000). Effects of excluded surface area and adsorbate clustering on surface adsorption of proteins: I. Equilibrium models.. Biophys Chem.

[58] Minton AP (2001). Effects of Excluded Surface Area and Adsorbate Clustering on Surface Adsorption of Proteins. II. Kinetic Models.. Biophys J.

[59] White DA, Buell AK, Knowles TPJ, Welland ME, Dobson CM (2010). Protein Aggregation in Crowded Environments.. J Am Chem Soc.

[60] Sun J, Weinstein H (2007). Toward realistic modeling of dynamic processes in cell signaling: Quantification of macromolecular crowding effects.. J Chem Phys.

[61] Rudan-Tasic D, Klofutar C (2003). Osmotic coefficients and solvation thermodynamics of aqueous solutions of some lower poly(ethylene glycol)s at different temperatures.. J Mol Liq.

[62] DiPaola G, Belleau B (1977). Polyol–Water interactions. Apparent molal heat capacities and volumes of aqueous polyol solutions.. Can J Chem.

[63] Bolen DW, Baskakov IV (2001). The osmophobic effect: natural selection of a thermodynamic force in protein folding.. J Mol Biol.

[64] Capp MW, Pegram LM, Saecker RM, Kratz M, Riccardi D (2009). Interactions of the Osmolyte Glycine Betaine with Molecular Surfaces in Water: Thermodynamics, Structural Interpretation, and Prediction of m-Values.. Biochemistry.

[65] Rosgen J, Pettitt BM, Bolen DW (2007). An analysis of the molecular origin of osmolyte-dependent protein stability.. Protein Sci.

[66] Athawale MV, Dordick JS, Garde S (2005). Osmolyte Trimethylamine-N-Oxide Does Not Affect the Strength of Hydrophobic Interactions: Origin of Osmolyte Compatibility.. Biophys J.

[67] Blodgett MB, Ziemer SP, Brown BR, Niederhauser TL, Woolley EM (2007). Apparent molar volumes and apparent molar heat capacities of aqueous adonitol, dulcitol, glycerol, meso-erythritol, myo-inositol, D-sorbitol, and xylitol at temperatures from (278.15 to 368.15) K and at the pressure 0.35 MPa.. J Chem Thermodyn.

[68] Eliassi A, Modarress H, Mansoori GA (1998). Densities of Poly(ethylene glycol) + Water Mixtures in the 298.15 K to 328.15 K Temperature Range.. J Chem Eng Data.

[69] Abramoff MD, Magelhaes PJ, Ram SJ (2004). Image Processing with ImageJ.. Biophotonics International.

[70] Hamley I, Castelletto V, Moulton C, Siligardi G, Oliveira C (2010). A Modified Amyloid Peptide Fragment Forming a Nematic Phase of Aligned Fibrils at Very Low Concentration.. Macromolecular Biosciences.

[71] Perczel A, Park K, Fasman GD (1992). Analysis of the circular dichroism spectrum of proteins using the convex constraint algorithm: A practical guide.. Anal Biochem.

